# Prevalence of Early Childhood Caries and the Related Factors among 3-5- Year-Old Children in Babol, Iran

**DOI:** 10.30476/DENTJODS.2021.88122.1313

**Published:** 2022-06

**Authors:** Moein Jamshidi, Mohammad Mehdi Naghibi Sistani, Neda Boushehri, Mahtab Hamzeh

**Affiliations:** 1 Dental Student, Student Research Committee, Babol University of Medical Sciences, Babol, Iran; 2 Dept. of Community Oral Health, Faculty of Dentistry, Babol University of Medical Babol, Iran; 3 Dept. of Pediatric Dentistry, Babol University of Medical Sciences, Babol, Iran

**Keywords:** Dental Caries, Prevalence, Risk Factors, Child, Preschool

## Abstract

**Statement of the Problem:**

Regular dental checkups to diagnose early childhood caries (ECC) are critical for maintaining and improving children's oral health and well-being. The prevalence of ECC is important for planning proper oral health programs.

**Purpose:**

This study aimed to investigate the prevalence of ECC and its related factors in children aged 3 to 5 years at Babol, Iran.

**Materials and Method:**

This cross-sectional study was conducted on 280 children aged 3-5 years in the kindergartens of Babol. Children were examined for dental caries according to World Health Organization criteria. The variables such as age, gender, health, nutritional habits, parents' educational level, parents' job status, and level of dental care were recorded in the questionnaire. Data were analyzed using t-test, chi-square and ANOVA and *p*< 0.05 was considered significant.

**Results:**

Average decayed-missing-filled teeth (dmft) were 4.03±3.6 and 73.2% in children with ECC, while 26.8% were caries-free. A significant association was found between age, snack consumption, father’s education, mother’s job, nocturnal drinking milk, tooth-brushing, and a history of dental check up before age 2 with ECC.

**Conclusion:**

The results of this analysis showed a high prevalence of ECC in Babol. Therefore, educational and interventional programs in prevention and dental health care, especially for mothers, nurses, and teachers of these age groups, should be considered.

## Introduction

Despite the significant improvement of oral health in the past few decades, dental caries is still among the most common chronic diseases of early childhood [ 1 ]. Different terms have been used to refer to the presence of dental caries among young children. Early childhood caries (ECC) is described as "the presence of one or more decayed tooth (non-cavitated or cavitated lesions), missing tooth (due to caries), or filled tooth surfaces in any primary tooth of a 72- month-old or a younger child”. In children younger than three years old, smooth-surface caries denotes severe early childhood caries (S-ECC). From ages 3 to 5, missing teeth (due to caries), one or more cavitated, or filled smooth surfaces in primary maxillary anterior teeth, or missing, decayed, or filled score of ≥4 (age 3), ≥5 (age 4), or ≥6 (age 5) surfaces constitutes S-ECC [ 2 ].

ECC is an early, moderate, and slow dental decay that affects infants and toddlers' primary teeth. It develops on tooth surfaces, which are regularly at low risk for caries. These include the labial surfaces of maxillary incisors or buccal and lingual surfaces of mandibular and maxillary molars [ 3 ]. This kind of dental caries initially appears as dull white or brown spots on maxillary incisors along the gingival margin, which progresses to the crown destruction, leaving root stumps [ 3 ]. In the moderate stage, caries starts to spread to maxillary molars. In the severe stage, the caries proceeding destroys maxillary teeth and reaches mandibular molars [ 2 - 3 ]. S-ECC may develop to “atypical”, “acute”“, progressive”, or “rampant” dental caries in children. A child with ECC may experience substantial pain, which may cause difficulty in eating and speech [ 3 ]. If the extent of the damage ends up to the extraction of the anterior teeth by age 2 or 3 years, the child may experience additional developmental delays involving speech articulation and patterns [ 3 ]. The consequences are delays in physical development concerning the poor nutrition, and the subsequent pain and discomfort may compromise their desire to eat. Therefore, the pain and suffering associated with caries affect the child's quality of life [ 3 ].

ECC incidence in different countries and even between different groups in society varies according to several predisposing factors [ 3 - 7 ]. These factors include frequent consumption of foods containing high sugar and the presence of *Streptococcus mutans* [ 3 ]. However, factors such as parental caries experience, low social class, low family income, and single-parent families are still being discussed [ 8 - 9 ]. The parents' education and social living conditions indicate the family's social level and may be associated with knowledge and skills related to oral health [ 4 , 7 ]. The impact of nutritional habits on oral health has been discussed in previous studies [ 10 - 13 ]. Some surveys indicate that breastfeeding and bottle-feeding are related to ECC development [ 10 - 11 ], while others have shown no association between these factors and caries incidence [ 12 - 13 ]. In addition, the influence of factors such as the age brushing started, brushing before bed, and brushing time on the prevalence of childhood caries is controversial [ 12 , 14 - 18 ].

Bagherian and Sadeghi [ 19 ] reported a prevalence of 51.2% dental caries in Iranian children in 2013. The severity of the disease slightly increased along with its pr-evalence and reached to 55% by 2014 [ 20 ] and in 2015, Hamissi [ 21 ] reported 68.1% with an overall mean dec-ayed-missing-filled teeth (dmft) score of 3.167 (±3.003).

Because of the controversial results of previous studies regarding the factors associated with ECC and the absence of a newly-published article about the prevalence of this disease in the north of Iran, this study was conducted to investigate the prevalence of ECC and its related factors in children 3-5 years old in Babol, Iran.

## Materials and Method

### Sampling and sample size

Stratified cluster random sampling served to examine 280 children 3-5 years old from nine kindergartens in Babol, Iran. Kindergartens were classified based on welfare rating (1 to 3 stars) and samples were randomly selected based on gender quotas and the population in these kindergartens. Based on the distribution of kindergartens in the city, 40, 50 and 10 percent of children were selected from 1, 2 and 3 stars' kindergartens. The distribution of boys and girls was equal in each group.

### Calibration of the Examiners

The principal researcher examined all studied samples. First, the examiner practiced examinations under a pediatric dentist (master) on a group of 10 children. Then, the examiner and the master examined the group of 20 children alone. This procedure was repeated, and findings were compared until an 85-95% agreement was obtained between the examiner and his master. This examination was repeated in the same group of 20 children a week later for reliability assessment. Results showed an 85 % agreement.

### Oral Examination

This cross-sectional study was carried out in the kindergartens of Babol for two months. A senior dental student using a disposable mirror in natural light did all clinical examinations. Before the test, the tooth surfaces were cleaned with sterile gauze. Senior dental student examined very young recruited children in the adjacent room in the knee-to-knee position. Intraoral examination to assess dental caries was done according to the World Health Organization (WHO) criteria [ 22 ]. To determine dmft, all filled, decayed, or extracted teeth (due to decay) were marked in a dental chart.

The examiner asked each child's mother to complete a questionnaire anonymously, including background information such as child’s age, birth weight, the child's eating habits, child's dental health behavior, parents’ education, and parents’ job status.

### Measures

Alternative responses for the frequency of tooth brushing were "once daily”, "twice or more daily," "sometimes," and "never," but for analysis, were dichotomized as "once and more daily” and “less than once daily” based on optimal tooth brushing [ 23 ]. We considered three categories, "Zero time/night”“, 1-2 times/night," and "> two times/night" for drinking milk during the night in the questionnaire. For analysis, we dichotomized it to "≤2 times/night", ">2 times/night".

Parents' education was categorized and scored into three levels as first level (both parents had a diploma, score 3), second level (one of them had a diploma, score 2) and third level (both parents had less than a diploma, score 1). In addition, parents' job was categorized and scored into three levels as first level (both parents were employed, score 3), the second level (one of them were employed, score 2), third level (both parents were unemployed, score 1). For analysis, we accumulated job and education scores and dichotomized these measures into high (summation of 5 and 6 scores) and low (summation of 2, 3 and 4) as families' socioeconomic status [ 24 ].

### Statistical Analysis

We double-checked all data by considering the original survey and examination forms to omit the data entry errors. Descriptive statistics, including frequencies, mean, median, and standard deviation, were carried out to obtain samples' overall characteristics and prevalence. We used t-test, chi-square, and ANOVA for analytical statistics. Significance was set at *p*< 0.05. We analyzed all data by using SPSS software for Windows (version 22).

The Ethical Committee of Babol University of Medical Sciences accredited this study (IR.MUBABOL. REC.1396.71). The examination date has been notified to the kindergartens' managers, and mothers were asked to be present on the examination date. Before the test, the study's goal was described for mothers and their consent was obtained. Participation in this investigation was voluntary.

## Results

Two hundred eighty children aged 3 to 5 years (140 boys and 140 girls) have been examined in this study. The mean (±SD) age was 4.45 (±0.71). We selected 28, 140, and 112 children from 3-, 2-, and 1- star kindergartens, respectively. The descriptive characteristics of the analysis sample are shown in Table 1. Overall, 205 (73.2%) children had ECC, while 75 (26.8%) were caries-free. According to the families' educational level and occupation status, higher ECC was associated with lower educated fathers (*p*< 0.05) and unemployed mothers (*p*< 0.001); however; regarding both parents' educational level and job status, the higher prevalence was found among children whose parents were in lower education and job level (*p*= 0.05) (Table 2).

**Table 1:** Descriptive characteristics of the study sample (n=280)

		Frequency	Valid percent
Parents’ education and job	Low	102	%36.4
	High	178	63.8%
Nocturnal milk-drinking	Zero time/night	11	3.9%
	1-2 times/night	105	37.5%
	>2 times/night	164	58.6%
Tooth-brushing	≥1/day	146	52.1%
	<1/day	134	47.9%
Responsible for brushing	Child	51	18.2%
	Parents	47	16.8%
	Child under the supervision of parents	182	65%
Dental examination before age 2	Yes	17	6.1%
	No	263	93.9%
Reason of visiting their dentists	Examination	47	28.7%
	Pain	54	32.9%
	Filling	49	29.9%
	Extraction	14	8.5%

**Table 2:** Prevalence of early childhood caries (ECC) and mean dmft by age, sex, parents’ education-job and kindergarten star (n=280)

	ECC			dmft	
	Yes	No	*p* Value^†^	Mean(SD)	*p* Value
**Age**					
3	17(45/9%)	20(54/1%)		1/68(±2/23)	<0.001*
4	55(69/6%)	24(30/4%)		3/28(±3/2)	
5	133(81/1%)	31(18/9%)	<0.001	4/93(±3/74)	
**Gender**					
Boy	99(70/7%)	41(29/3%)		3/96(±3/68)	0.8^γ^
Girl	106(75/7%)	34(24/3%)	0.2	4/1(±3/56)	
**Parents 'education and job**					
Low	81(79/4%)	21(20/6%)		4/53(±3/69)	0.6
High	124(69/7%)	54(30/3%)	0.05	3/75(±3/55)	
**Kindergarten star**					
1	78(69/6%)	34(30/4%)		4/4(±4/11)	0.3
2	107(76/4%)	33(23/6%)		3/84(±3/11)	
3	20(71/4%)	8(28/6%)	0.4	3/51(±3/8)	

Mean (±SD) dmft was 4.03 (±3.6) and mean (±SD) of each part as d (decay), m (missing) and f (filling) were 3.1 (±0.32), 0.2 (±0.82) and 0.72 (±1.48), respectively. The systemic disease was evident among 34 (12.1%) children, which the most popular one was Enzyme deficiency and 21(7%) studied children who used drugs of which ferrous sulfate and levothyroxine were most popular. Table 3 shows the relationship between ECC and the last dental visit. The lowest dmft was visible among children who visited their dentist for a check-up (*p*< 0.001).

**Table 3:** Early childhood caries( ECC) and dmft by dentistry experience of children (n=280)

	ECC			Dmft	
	Yes	No	*p* Value	Mean(SD)	*p* Value
**Dental examination before age 2**					
Yes	9(52/9%)	8(47/1%)		3/36(±3/57)	0.3
No	196(74/5%)	67(25/5%)	0.05	4/76(±7/76)	
**Reason of visiting their dentist**					
Examination	54(100%)	0(0/0%)		1/66(±2/61)	<0.001
Pain	49(100%)	0(0/0%)		5/3(±2/43)	
Filling	21(44/7%)	26(55/3%)		6/43(±3/2)	
Extraction	10(71/4%)	4(28/6%)	<0.001	8/14(±5/9)	

Regarding feeding habits, the prevalence of “breastfeeding," “bottle feeding" and “breast and bottle feeding were 204 (72.9%), 19 (6.8%) and 57(20.4%), respectively. As reported by parents, 259 (92.5%) children used sugary snacks (biscuit, chocolate, cake) between meals. Higher ECC was shown in the children who consumed snacks between meals (*p*< 0.001), fed milk more than two times during the night (*p*= 0.02), and brushed less than once daily (*p*<0.001) (Table 4).

**Table 4:** ECC and dmft by feeding and oral hygiene behavior of children (n=280)

	ECC			dmft	
	Yes	No	*p* Value	Mean(SDI)	*p* Value
**Snack consumption**					
Yes	197(76/1%)	62(23/9%)		4/15(±3/57)	0.3
No	8(38/1%)	13(61/9%)	< 0.001	2/62(±3/94)	
**Nocturnal milk-drinking**					
≤2 times/night	76(65/5%)	40(34/5%)		3/55(±3/77)	0.7
>2 times/night	129(78/7%)	35(21/3%)	0.02	4/37(±3/47)	
**Tooth-Brushing**					
≥1/day	91(62/3%)	55(37/7%)		3/36(±3/57)	0.3
<1/day	144(85/1%)	20(14/9%)	< 0.001	4/76(±3/54)	
**Responsible for Brushing**					
Child	43(84/3%)	8(15/7%)		5/12(±3/78)	0.01
Parents	28(59/6%)	19(40/4%)		2/96(±3/79)	
Child under the supervision of parents	134(73/6%)	48(26/4%)	0.02	4/01(±3/45)	

## Discussion

This survey was performed to evaluate early caries prevalence and severity in 280 children in a city in Northern Iran and identify the factors related to this condition. The prevalence in this study was 73.2 %. Previous surveys conducted in different parts of Iran have reported various lower prevalence values, as 55% in Shiraz [ 20 ], 51.2% in Rafsanjan [ 19 ], 63.4% in Shemiranat [ 25 ], and 68.1% in Qazvin [ 21 ].

Inconsistent with previous surveys [ 26 - 27 ], we found a significant association between the child's higher age and the higher ECC prevalence. With the increase in age, the duration of exposure of teeth to cariogenic factors increases. Therefore, it is rational to yield higher prevalence in older children.

We found a higher incidence of ECC among girls. Conversely, in the study performed by Koya *et al*. [ 28 ], the prevalence was higher in boys. However, Amanlou *et al*. [ 29 ] and Toutouni *et al*. [ 30 ] in Iran found no significant relationship between gender and ECC.

In the current study, the prevalence of early caries in children of higher socioeconomic class families was significantly higher than that in lower socioeconomic class ones. Casanova-Rosado *et al*. [ 23 ] and Amanlou *et al*. [ 29 ] reported similar findings. An investigation carried out by Popoola *et al*. [ 31 ] showed that the average number of dental caries in children with higher socioeconomic status seems to be more. In our study, the employment status of parents was considered as a socioeconomic determinant. Higher income can be related to more access to media and scientific researches, and higher education leads to more understanding of oral hygiene instructions. These factors collectively may lead to a lower prevalence of ECC in families with a more upper social class.

American Academy of Pediatric Dentistry guidelines advocate that all children have their initial dental visit during the first year of life [ 32 ]. Our findings showed that children who had a dental examination before the second year of their life had a lower average dmft. Parents who take their children for regular dental visits are better informed about oral hygiene instructions. If early carious lesions are diagnosed and treated by dentists during these regular checkups, the oral environment will be less susceptible to dental caries in future. 

Similar to previous findings [ 33 - 35 ], the higher incidence of early caries was evident among those children who had the habit of drinking or eating sweets after dinner every day, and the intakes of candy, soda, or isotonic drinks were more than four days a week. 

ECC was related to bottle-feeding and sleeping with a bottle [ 27 , 33 , 36 ]. We also observed that breastfeeding and frequency of nocturnal milk-drinking increased the number of ECC and the mean dmft in children. Some case report studies suggested that prolonged and excessive breastfeeding is associated with rampant tooth decay in infants [ 37 - 38 ]. There appears to be a clinical consensus between dental practitioners that prolonged and nocturnal breastfeeding is associated with an increased ECC risk, especially after 12 months of age. The controversial issue of the cariogenecity of human milk is still unresolved [ 39 ].

Adequate oral hygiene is one of the necessities for avoiding childhood caries. However, in the present study, the vast majority of the children who exhibited poor oral hygiene were characterized by the presence of clinically visible plaque. Higher dental caries was associated with lower supervision during tooth-brushing, revealing insufficient preschoolers' manual skills to maintain adequate oral hygiene [ 14 , 27 ]. Similarly, our results showed that early caries decreased when parents were responsible for children's tooth brushing, and they were doing this duty at least once a day.

To note the limitations of the study, we employed a self-administered questionnaire to record all potential associated factors. Hence, many families preferred not to give detailed information, especially about their job status, for personal considerations. Besides, due to the limited sample size, the classification of university education was difficult and unreliable. As with any study carried out on a self-administered questionnaire, the information resulting from parents' memory may not be completely accurate and might have recall bias. 

## Conclusion

The results of this study showed a high prevalence of ECC in Babol. We found that poor oral hygiene, low socioeconomic status, consuming sweet snacks, and breastfeeding more than twice per night are related to higher early caries. Rigorous preventive oral health programs should be planned for children less than five years old, especially for lower socioeconomic class families.

## Conflict of Interest

The authors declare that they have no conflict of interest.
